# Web-Delivered Multimedia Training Materials for the Self-Collection of Dried Blood Spots: A Formative Project

**DOI:** 10.2196/11025

**Published:** 2018-11-05

**Authors:** Alicia M Allen, Kim Lundeen, Sharon E Murphy, Logan Spector, Bernard L Harlow

**Affiliations:** 1 Department of Family & Community Medicine College of Medicine University of Arizona Tucson, AZ United States; 2 Department of Family Medicine & Community Health Medical School University of Minnesota Minneapolis, MN United States; 3 Division of Biochemistry Molecular Biology and Biophysics University of Minnesota Minneapolis, MN United States; 4 Division of Epidemiology and Clinical Research Pediatrics University of Minnesota Minneapolis, MN United States; 5 Department of Epidemiology School of Public Health Boston University Boston, MA United States

**Keywords:** dried blood spot, internet, feasibility studies

## Abstract

**Background:**

The use of dried blood spots (DBS) in biomedical research has been increasing as an objective measure for variables that are typically plagued by self-report, such as smoking status and medication adherence. The development of training materials for the self-collection of DBS that can be delivered through the Web would allow for broader use of this methodology.

**Objective:**

The objective of this study was to evaluate the acceptability and feasibility of the self-collection of DBS using newly developed multimedia training materials that were delivered through the Web. We also aimed to assess the usability of the collected DBS samples.

**Methods:**

We recruited participants through Facebook advertising for two distinct studies. The first study evaluated the acceptability of our newly developed DBS training materials, while the second assessed the implementation of this protocol into a larger Web-based study.

**Results:**

In the first study, participants (N=115) were aged, on average, 26.1 (SD 6.4) years. Training materials were acceptable (113/115, 98.2%, of participants were willing to collect DBS again) and produced usable samples (110/115, 95.7%, collected DBS were usable). In the second study, response rate was 25.0% (41/164), with responders being significantly younger than nonresponders (20.3 [SD 0.2] vs 22.0 [SD 0.4]; *P*<.001), and 92% (31/41) of collected DBS samples were usable by the laboratory.

**Conclusions:**

Overall, while the protocol is acceptable, feasible, and produced usable samples, additional work is needed to improve response rates.

## Introduction

Dried blood spots (DBS) are drops of whole blood that are collected on filter paper from a finger prick [1]. A wide range of biomarkers have been validated for assessment in DBS (Multimedia Appendix 1) [1-10]. The number of DBS biomarkers surpasses the number of validated biomarkers in urine or saliva samples alone [1,11]. The use of DBS in biomedical research has been growing as it allows objective measurement of variables that have been troubled by self-report and recall bias (eg, biological confirmation of smoking status or medication compliance) [1]. Furthermore, the Centers for Disease Control and Prevention has stated that DBS “...has achieved the same level of precision and reproducibility that analytical scientists and clinicians have come to expect from standard methods of collecting blood such as vacuum tubes...” [12]. In addition to the wide range of applicable uses, DBS also provides other advantages over venipuncture and other biospecimen collection methods. DBS samples are stable at room temperature for a longer time period, and their collection is less costly and less burdensome for study participants [1,11].

A newly demonstrated benefit of biomarker analysis using DBS is the ability for the self-collection of samples by study participants. Successful self-collection of DBS has been reported in a wide variety of study samples from children with epilepsy [13] to individuals at high risk for HIV [14] and diabetics [15]. These studies have demonstrated high accuracy of collection as well as high satisfaction with the collection process. For instance, in a sample of adults with diabetes, 94% collected DBS correctly, and 97% reported that collection was easy [15]. Similarly, while 32% of individuals at high risk for HIV reported some discomfort with DBS collection, 92% reported that the process was easy and 93% indicated a willingness to collect DBS in the future [14].

Unfortunately, the use of DBS methodology is limited as nearly all studies have relied on in-person training for the self-collection of DBS. However, Roberts et al [4] recently utilized the self-collection of DBS after training via a printed brochure that was delivered through the mail. While 91% of their samples were usable, they recommended the use of a training video to reduce the number of suboptimal DBS specimens. We sought to eliminate the need for in-person training of accurate DBS self-collection by creating standardized written and video training materials that could be delivered through the Web. Therefore, the goal of this project was to evaluate the acceptability, feasibility, and usability of DBS samples that were collected after utilizing newly developed multimedia training materials delivered through the Web. To address this goal, we conducted two studies. In Study 1, we tested the newly developed training materials to determine the acceptability of the self-collection of DBS after exclusive Web-based training, as well as the usability of DBS samples. In Study 2, we implemented the DBS protocol within a larger ongoing study to assess the feasibility of our protocol. Eliminating the in-person training, in favor of Web-delivered training, will substantially reduce the logistical hurdles involved with collecting DBS samples. This will allow DBS collection to occur without any in-person interaction between study staff and participants, thereby allowing DBS to be collected with relative ease and be used for biomarker analysis in large, geographically diverse, population-based studies.

## Methods

### Development of Training Materials

To address our study goal, we created a video with corresponding written training materials. These items were modeled after previously created materials that were used to train medical professionals for the collection of DBS [11] and those that have been previously used for in-person training for DBS collection in an ongoing HIV-related research project at Emory University (personal communication with Dr AD Mc Naughten, March 11, 2014; August 12, 2015; September 3, 2015). Initially developed materials were qualitatively reviewed by a small local sample (n=20) of male and female cigarette smokers (data not shown). After the identification of two major gaps in the training materials (clear instructions to not touch the filter paper and what to do if blood is not flowing enough), the training materials were revised. Final training materials can be found in Multimedia Appendices 2 and 3. All study-related procedures were approved by the institutional review board at the University of Minnesota.

### Study 1

#### Recruitment

Upon completion of the training materials, we recruited a convenience sample of study participants through advertising on Facebook. Recruitment advertising occurred for 1 week in April 2016 and was restricted to individuals in the United States between the ages of 18 and 35 years. After clicking on the advertisement, eligibility was assessed through a Research Electronic Data Capture (REDCap) survey [16]. To be eligible for the study, participants had to be between the ages of 18 and 35 years and self-report being a daily smoker at a rate of at least 5 cigarettes per day. These inclusion criteria were selected given a secondary goal of examining the relationship between sex hormones (eg, progesterone) and smoking-related biomarkers (eg, cotinine). Potential participants were excluded if they reported having difficulties (eg, feeling nauseous or dizzy) with the sight of blood.

#### Study Procedures

Upon confirmation of eligibility, participants provided informed consent and completed a pretest survey. In addition to information regarding demographics and smoking behavior, the pretest survey included 3 questions to assess expectations regarding expected difficulties with DBS collection, as well as confidence and willingness to complete DBS collection. Participants responded using a 100-point visual analog scale ranging from “very easy” or “not at all” to “very hard” or “very willing.” In addition, we asked if participants had any prior experience with DBS collection (yes, no, not sure).

Next, participants viewed the 5-minute training video. We recorded the length of time the webpage displaying the video was open. Upon completion of the video, participants completed a posttest survey, which contained 3 sections. The first section was video response section in which participants answered 6 questions with 4 options (ranging from “strongly agree” to “strongly disagree”) regarding the clarity of the video. The next section was the knowledge section that contained 6 true or false statements regarding the proper way to collect DBS (eg, “It is okay to touch my finger to the collection card”). The last section of the posttest survey assessed the willingness to collect DBS. Participants were asked if they would be willing to collect DBS and mail the DBS sample to the University of Minnesota in exchange for a US $25 Amazon or Target e-gift card. Those who indicated they would not be willing to collect were asked why they did not want to collect DBS. Those who indicated they would be willing to collect were routed to a survey in which they provided their mailing address. All data collection was completed through REDCap [16].

Finally, DBS collection materials were mailed to participants. The mailing included a letter, which contained a URL link to the training video, as well as a printed copy of the written instructions and placemat. Three DBS collection kits were sent to each participant—one kit, along with two back-ups to be used if necessary. Each kit contained 1 micro-lancet, 1 collection card (a 903 Protein Saver Card purchased from GE Healthcare Life Sciences), 2 gauze pads, 1 alcohol wipe, and 1 bandage. Furthermore, a postcollection survey was included to assess the overall impression of the process as well as the impression of the video and written materials. Additional materials included a pen, packaging materials (a desiccant packet, humidity indicator, and plastic biohazard bag), and a prepaid return envelope. The cost of supplies for each collection kit was US $7.56 plus US $5.34 in postage for a total of US $12.90 per mailing.

We defined feasibility as the number of DBS samples received back from study participants. The laboratory staff then classified samples into one of four categories: excellent, satisfactory, poor, and not usable.

### Study 2

#### Recruitment

From July to September 2016, we recruited a new and larger convenience sample including current smokers (defined as a self-report of smoking ≥100 cigarettes in their lifetime and smoking on at least 4 of the past 30 days) in the United States between the ages of 18 and 35 years through Facebook advertising to assess the role of hormonal contraceptive on smoking-related behaviors (results forthcoming). Per the goals of the parent project, participants were selected for this ancillary DBS project as follows: (1) all female participants who reported current use of any of the following types of hormonal contraceptives: Implanon (n=14), Mirena intrauterine device (n=17), or combination oral contraceptives with a stable daily ethinyl estradiol dose between 20 and 30 micrograms (n=43); (2) a simple random sample of 45 female participants who reported regular menstrual cycles and no use of exogenous hormones; and (3) a simple random sample of 45 male participants.

#### Study Procedures

Eligible participants were emailed an invitation to participate in this ancillary study, which contained a link to view the DBS training video as well as a link to a survey on REDCap [16], where interested participants would provide informed consent and their mailing address. After the original email invitation, eligible participants received an additional three reminder emails to participate in the ancillary study on 3, 6, and 14 days after the original invitation email was sent. All data regarding demographics and smoking behavior were collected from study participants through REDCap prior to the invitation to this ancillary study. All participants were paid a US $10 Amazon e-gift card for completion of this 20-minute survey. Participants who expressed an interest in completing the DBS protocol were sent the same materials as described above. Due to time restrictions with the study grant, participants were given 1 month to return completed DBS samples in exchange for a US $50 Amazon e-gift card. Email reminders were sent 2 weeks and 5 days prior to the collection deadline.

### Statistical Analysis

Descriptive statistics (means and SEs for continuous variables and percentages and counts for categorical variables) were computed to describe the demographics and smoking behavior of the study sample separately for both studies. In addition, descriptive statistics were computed for the following items collected in Study 1: (1) expectations from the pretest survey in Study 1; (2) the clarity of the video, knowledge of the process, and willingness to collect DBS from the posttest survey; and (3) receipt of DBS samples, responses to postcollection survey, and laboratory staff rankings of “usability.” In both studies, simple *t* tests and chi-square tests were performed to compare the demographics and smoking behavior between those who were willing to collect DBS versus those who were not, as well as those who returned their DBS samples versus those who did not. All analyses were conducted using SAS 9.3 (SAS Institute).

## Results

### Study 1

#### Study Sample

A total of 242 participants provided informed consent and completed the eligibility survey. A total of 28 were excluded due to the following reasons (items are not mutually exclusive): age (n=12), smoking <5 cigarettes per day (n=21), or not being comfortable working with blood (n=9). Therefore, of all the participants enrolled into the study, 53.7% (115/214) completed the Web-based survey. The 115 participants who completed the survey were on average 26.1 (SD 6.4) years of age and smoked 17.4 (SD 9.7) cigarettes per day. Most (107/115, 93.0%) participants were white people and approximately half had at least some college education (58/115, 50.4%); 50.4% (58/115) participants were male and 49.6% (57/115) smoked their first morning cigarette within 40 minutes of waking. The sample was also geographically diverse (using the US Census definition)—17.4% (2/115) of participants from the West, 37.4% (43/115) from the Midwest, 20.0% (23/115) from the Northeast, and 25.2% (29/115) from the South. No significant differences were observed between those who completed the study (n=115) and those who did not (n=99) in terms of demographics or smoking behavior.

#### Previewing Survey

Prior to viewing the training video, 35.7% (41/115) participants indicated that they had some prior experience with the self-collection of DBS. On average, participants did not think the DBS self-collection would be difficult (25.1 [SD 24.8] on a 100-point visual analog scale with “0” indicating “not at all difficult”) and were confident that they would be able to successfully self-collect DBS (74.5 [SD 25.68] on a 100-point visual analog scale with “100” indicating “very confident”). Upon completion of this survey, participants watched the training video. On average, the 5-minute video was viewed for 3.6 (SD 3.5) minutes, with 50.4% (58/115) of participants watching through the end of the instructions (4:38 minutes or more).

#### Postviewing Survey

After viewing the training video, most replied with “agree” or “strongly agree” to the following statements: (1) the instructions in the video were clear (113/115, 98.2%); (2) the video was enjoyable to watch (98/115, 85.2%); (3) after watching the video, I felt like I knew how to collect DBS (114/115, 99.1%); (4) after watching the video, I felt excited to collect DBS (89/115, 77.3%); (5) after watching the video, I felt more confident about my abilities to collect DBS (113/115, 98.2%); and (6) after watching the video, I felt more willing to collect DBS (104/115, 90.4%). As displayed in Table 1, most participants responded to the true or false questions correctly.

A total of 106 participants (106/115, 92.1%, of those who completed the survey; 106/214, 49.5%, of the initially enrolled sample) indicated they would be willing to self-collect DBS for this study and 86.9% (100/115) provided us with a mailing address to send the collection materials. Among those who declined participation, 8 participants provided a reason for declining, which included (item not mutually exclusive) wanting more compensation (6/8, 75%), feeling uncomfortable sending blood to researchers (2/8, 25%), and feeling uncomfortable sending identifying information, such as mailing address, to researchers (2/8, 25%).

#### Usability of Dried Blood Spot Samples

Of all DBS kits mailed out, we received 51.0% (51/100) DBS samples back for analysis. A total of 96% (49/51) DBS samples were rated as “usable” by the laboratory staff. Two samples were rated as not usable because the drops of blood on the card were too small.

**Table 1:** Knowledge questions with participant response (n=115).

Question	Response, n (%)	
	True	False
The blood spots should fill most the collection circle.	113 (98.2)^a^	2 (1.7)
It is okay to touch my finger to the collection card.	110 (95.6)^a^	5 (4.3)
If my drop of blood does not fill the collection circle, I should add a second drop of blood.	22 (19.1)	93 (80.7)^a^
It is okay if my blood drop falls slightly outside the collection circle.	92 (80.0)^a^	23 (20.0)
If my blood stops flowing and I haven’t filled all of the circles, I should prick another finger to try to fill all the circles.	67 (58.2)^a^	48 (41.7)
I should make sure the blood spots have dried for at least 4 hours before packaging the card.	109 (94.7)^a^	6 (5.2)

^a^The correct response.

**Table 2:** Postcollection survey (n=46).

Items and responses		Instructional booklet, n (%)	Video, n (%)
**The instructions in the (instructional booklet or video) were clear.**			
	Strongly agree	44 (93)	41 (89)
	Agree	2 (7)	4 (9)
	Disagree	0 (0)	1 (2)
	Strongly disagree	0 (0)	0 (0)
**After (reading the instructional booklet or watching the video) I felt more willing to collect dried blood spots.**			
	Strongly agree	33 (71)	29 (63)
	Agree	13 (29)	16 (35)
	Disagree	0 (0)	1 (2)
	Strongly disagree	0 (0)	0 (0)
**After (reading the instructional booklet or watching the video) I felt confident about my abilities to collect dried blood spots.**			
	Strongly agree	41 (89)	38 (82)
	Agree	5 (11)	7 (16)
	Disagree	0 (0)	1 (2)
	Strongly disagree	0 (0)	0 (0)

Among those who returned their DBS samples, 90% (46/51) also returned the postcollection survey. As displayed in Table 2, participants thought the instructional materials were clear, increased willingness to collect DBS, and invoked feelings of confidence. Agreement with these items was slightly higher for the instructional booklet versus the video. Furthermore, these survey results indicated that most participants were “definitely” (41/46, 89%) or “probably” (4/46, 9%) willing to collect DBS again.

### Study 2

#### Implementation

The email invitation was sent to a total of 164 eligible participants. Of them, 43.2% (71/164) provided informed consent and a mailing address. The nonresponders (n=93) were younger (20.3 [SD 0.2] vs 22.0 [SD 0.4]; *P*<.001), smoked on fewer days in the last 30 days (23.0 [SD 0.9] vs 25.9 [SD 0.8]; *P*=.01), and were more likely to be nonwhite people (25/93, 27%, vs 7/93, 7%; *P*<.001). There were no differences between responders and nonresponders in terms of gender, education, income, or cigarettes smoked per day.

Of the 71 DBS collection kits that were mailed to participants, 58% (41/71) returned DBS samples; 6% (4/71) kits were returned due to mailing or addressing errors and 37% (26/71) were not returned. Compared with those who returned the DBS kit (n=41), those who did not (n=30) were younger (22.9 [SD 0.5] vs 20.9 [SD 0.5]; *P*=.01). There were no differences in terms of gender, education, income, work status, race, ethnicity, or smoking behavior, including cigarettes smoked per day. Of the 41 samples received, 92% (38/41) were usable by the study laboratory.

## Discussion

### Principal Findings

Overall, the results of this project indicate that Web-delivered training on the self-collection of DBS was acceptable to study participants and that the protocol was feasible to implement. Specifically, the vast majority of participants indicated that both the instructional video and written materials increased their confidence and willingness to self-collect DBS. Furthermore, nearly all participants who self-collected DBS indicated they would probably or definitely be willing to collect again. Finally, nearly all self-collected DBS samples received were deemed usable by the laboratory staff. However, upon implementation of this protocol into a larger, ongoing study, our response rates remained low, despite increasing the financial incentives. This was especially true among the participants who were younger and of a racial minority.

Our observations regarding the usability of self-collected DBS are comparable to prior studies that used in-person training. For instance, in a study conducted by Fokkema et al [15] with an adult sample of diabetics who received in-person training on the self-collection of DBS, 95% of samples were collected correctly. Our laboratory staff determined that 95% (87 of 92) of our samples were collected correctly, which was slightly higher than the 91% rate observed in a recently published work that used written training materials only [4]. Furthermore, the prior literature on self-collection of DBS that used in-person training reported high rates of willingness to self-collect DBS again (eg, 93% in a sample of HIV-infected individuals [14]). We reported that 98% (41/46) participants were “definitely” and 9% (4/46) were “probably willing to self-collect DBS again. Therefore, our Web-delivered training materials led to similar success and acceptability rates compared with in-person training.

Notably, only half of the study sample viewed the training video through the completion of the instructions. While participants who received the DBS self-collection kits through the US mail were instructed to view the video a second time prior to collection, it is unknown whether or not participants actually did this and, if so, how long they watched the video. Compared with the video, the written materials were indicated to be clear, as well as increasing the willingness and confidence to self-collect DBS, by more participants. Given that 95.7% (110/115) of DBS samples received by us were usable by the laboratory, suggests that written training materials may be more informative than the video. Overall, these observations suggest that the written training materials may be adequate on their own without the training video. However, we did not formally compare the usefulness of these materials.

Unfortunately, we observed a response rate of approximately 25.0% (41/164) when implementing this protocol into a larger, ongoing Web-based study; this was despite doubling the incentive for returning collected DBS samples from US $25 to US $50 from Study 1 to Study 2, which was the most common reason for declining in Study 1. There were a number of significant differences between responders and nonresponders; the latter were younger and more likely to be nonwhite people. Our response rate is quite a bit lower than that observed in a recently completed project with young adult cancer survivors, which paid participants US $20 for completion [4]. It is likely that young adult cancer survivors are more motivated to participate in research than young adult smokers in the general population. Furthermore, the young adult cancer survivors were older and more educated than our sample. This suggests that in order to improve overall response rate, additional development in the protocol to encourage motivation for compliance is needed and study should be tailored to those who are younger or of a racial minority. For example, additional reminders (eg, via email, US mail, or texting) and more collection kits could be sent out to participants and a longer time for return of study samples could be provided. It may also be helpful to provide a small amount of initial compensation along with the mailed DBS collection kits to increase the incentive to return the completed DBS samples.

Although this study is the first to assess the feasibility of Web-delivered multimedia instructions for the self-collection of DBS, it is not without limitations. First, slightly less than half (99/214, 46.2%) of our participants discontinued the Web-based survey early in Study 1. Although those who discontinued the survey early were statistically comparable to those who completed the survey in terms of their demographics and smoking behavior, it is possible that selection bias is present in this study. Second, we limited our sample to individuals who were between the ages of 18 and 35 years and were daily cigarette smokers. Therefore, it is unknown whether our observations are generalizable to other populations. Third, there are ethical considerations that need to be taken into account when performing research on Facebook (eg, verification of the age of consent). Next, in Study 2, we had a somewhat limited timeline due to funding restrictions. It is possible that our response rates would have been better if we allowed participants to return their DBS at a later date. Finally, we did not directly compare our training materials (eg, video and training booklet) to each other nor did we use a control group. Therefore, it is unknown which materials are driving our observations. These methodologies would be strengthened with this type of comparison; thus, future research should pursue this.

### Conclusions

These newly developed multimedia training materials for the self-collection of DBS are acceptable to study participants, feasible to implement within a Web-based setting and yield usable self-collected DBS. However, response rates were low. Therefore, additional work is needed to improve response rates, especially among certain subgroups of the population. Future research pursuing improving the response rates would allow for the elimination of in-person training and, therefore, substantially expand the application of DBS to a variety of areas such as telemedicine, large population-based epidemiology studies, and Web-delivered intervention studies.

## Multimedia Appendix 1

Biomarkers measurable in dried blood spots.

## Multimedia Appendix 2

Instructional video.

## Multimedia Appendix 3

Written materials including the instructional booklet (3A) and placemat (4B).

## Abbreviations

DBS: dried blood spots
REDCap: Research Electronic Data Capture
