# Supplementary material for: Web-Delivered Multimedia Training Materials for the Self-Collection of Dried Blood Spots: A Formative Project
Source: JMIR Form Res. 2018 Nov 5;2(2):e11025. doi: 10.2196/11025 (PMC6334672; doi:10.2196/11025)
Supplement: Supplementary file 1 [file formative_v2i2e11025_app1.pdf]

Multimedia Appendix 1. Biomarkers measureable in dried blood spots

- 11-deoxycortisol
- 17α-hydroxypregnenolone
- 17α-hydroxyprogesterone
- 17-dione
- 21-deoxycortisol
- 25-hydroxyvitamin D3
- 3'-hydroxycotinine
- 4-androstene-3
- α1-antitrypsin genotypes
- Acid beta-D-glucosidase
- Acid sphingomyelinase
- Acylcarnitines
- Adenosine-deaminase defect
- Adrenal steroids
- α-galactosidase
- α-iduronidase
- Alloisoleucine
- Alpha-N-acetyl-galactosaminidase
- Androstenedione
- Anti-Mullerian Hormone
- Apolipoprotein A-I
- Apolipoprotein B
- Babesiagibsoni Infection
- Beta-Carotene
- Biotinidase
- Bisphenol A (BPA)
- C26:0-lysophosphatidylcholine
- Carnitine and Acylcarnitines
- Cd4+ lymphocytes
- Cd4+ t-cell
- Ceruloplasmin
- Chitotriosidase
- Citrulline
- Corticosterone
- Cortisol
- Cotinine
- C-reactive protein
- Creatine
- Cytomegalovirus infection
- Dehydroepiandrosterone-sulfate
- Deoxycorticosterone
- Dermatansulfate
- DHEA-S
- Dihydrotestosterone
- Epstein-Barr Virus Antibodies
- Estradiol
- Folate
- Follicle-Stimulating Hormone
- Frataxin
- Gamma-Hydroxybutyric Acid
- Gaucher Disease
- Glucocerebrosidase
- Glucose
- Gluthathione
- Guanidinoacetate
- H. influenzae b
- Hemoglobin
- Hemoglobin A1c
- Heparinsulfate
- Hepatitis A Antibodies
- Hepatitis B Antibodies
- Hepatitis B Antigen
- Hepatitis C Antibodies
- HIV Antibodies
- Homocysteine
- HTLV-I specific antibodies
- Human Chorionic Gonadotropin Hormone (free B subunit)
- Hunter syndrome
- IFNg
- IgE
- IGFBP-2
- IGFBP-3
- IGF-I
- IL-1b
- IL-6
- Inflammatory markers
- Insulin
- Interferon-γ inducible protein (IP)-10
- Interleukin-6
- Lipoprotein (LP)
- LSD Enzyme
- Luteinizing Hormone
- Lymphoproliferative diseases
- Mucopolysaccharidosis type II
- Neurotrophins
- Perfluorooctanesulfonate (PFOS)
- Perfluorooctanoate (PFOA)
- Phenylalanine
- Phenylketonuria
- Phosphatidylethanol
- Pompe disease
- Pregnancy-Associated Plasma Protein A
- Progesterone
- Prolactin
- Prostate-Specific Antigen (PSA)
- Retinol
- S. pneumoniae
- Sandhoff Disease
- Sex Hormone Binding Globulin
- Somatomedin-c
- Succinylacetone
- Tay-sachs Disease
- Testosterone
- Thyroglobulin
- Thyroid Antibody
- Thyroid Stimulating Hormone (TSH)
- Thyroxine (T4)
- Thyroxine Binding Globulin
- TNFa
- Tocopherols
- Trans-3'-hydroxycotinine
- Transferrin Recepter
- Triglycerides
- Triiodothyronine (T3)
- Tyrosine
- Vitamin C
- Vitamin D2-25-OH
- Vitamin D3-25-OH

Compiled from references 1-10.
