# Supplementary material for: Web-Delivered Multimedia Training Materials for the Self-Collection of Dried Blood Spots: A Formative Project
Source: JMIR Form Res. 2018 Nov 5;2(2):e11025. doi: 10.2196/11025 (PMC6334672; doi:10.2196/11025)
Supplement: Supplementary file 3 [file formative_v2i2e11025_app3.pdf]

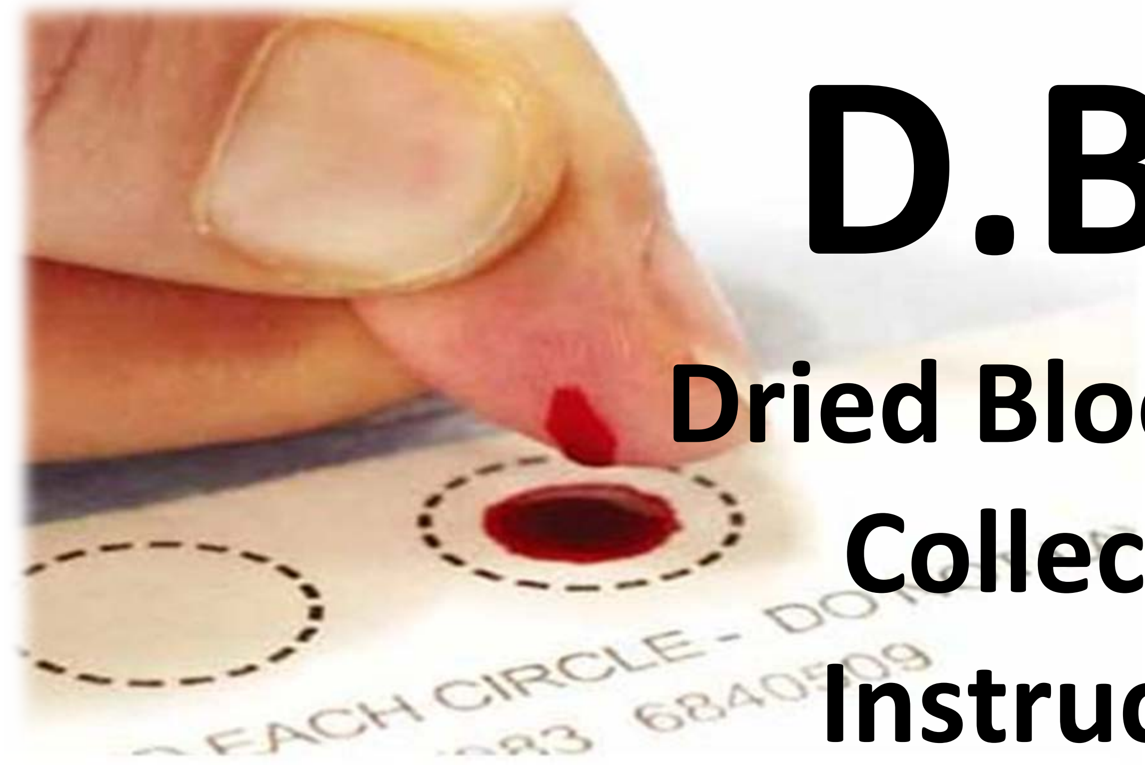

D.B.S.

Dried Blood Spot Collection Instruction Booklet

With Information Provided By:

The University of Minnesota

Department of Family Medicine and Community Health

A. SET-UP

Step 1

Before you begin, make sure you will have at least 30 minutes of uninterrupted time.

If you have questions, please call (612-624-5081), text (612-213-5864) or email (dbs@umn.edu) us!

First, find a clean, open space to work on. Lay out all of your materials on the placemat provided.

The materials in your kit:

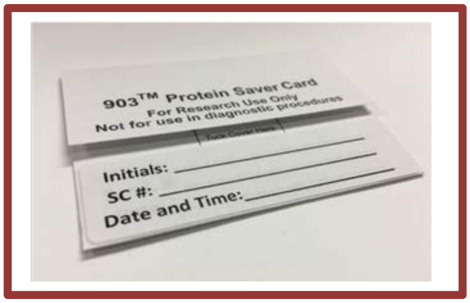

Blood specimen collection card

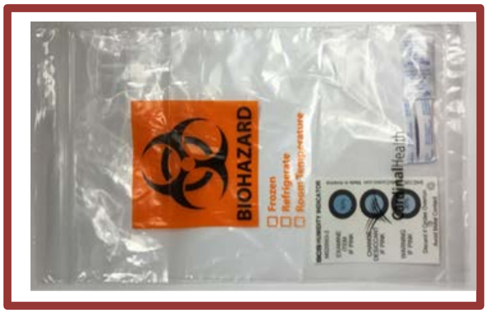

Packaging materials

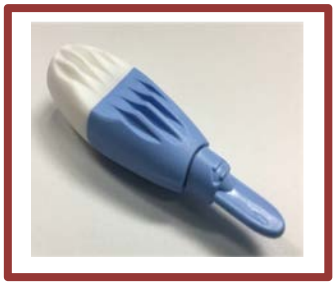

Safety lancet

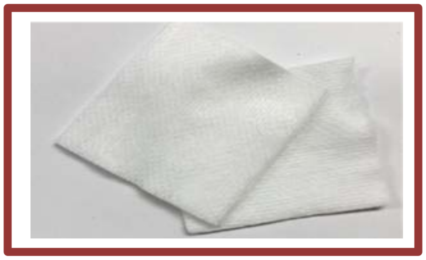

Gauze pads

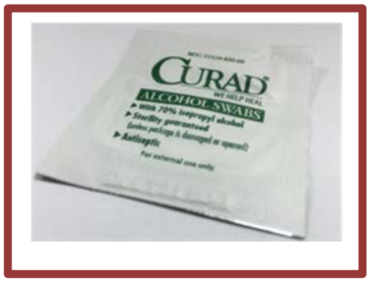

Alcohol wipe

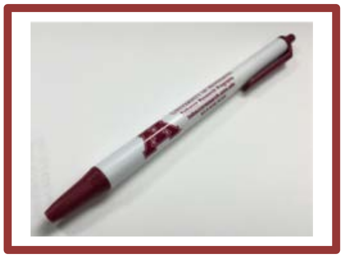

Pen

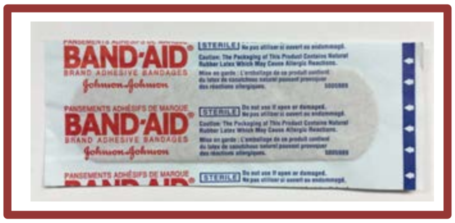

Bandage

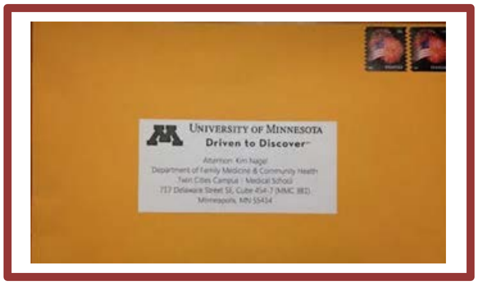

Return envelope

Please note that your packet also includes two additional blood collection kits that you can use if necessary.

Step 2

Write the date and time on the blood specimen collection card.

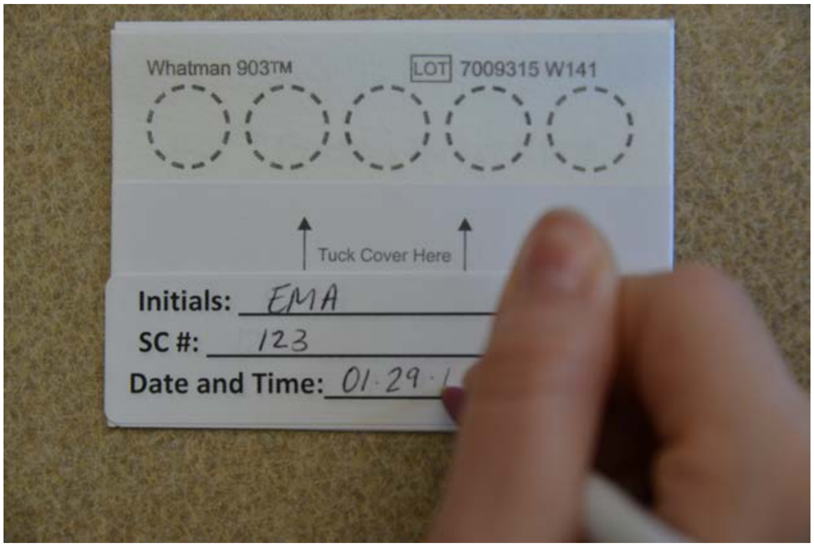

Step 3

Open the alcohol wipe.

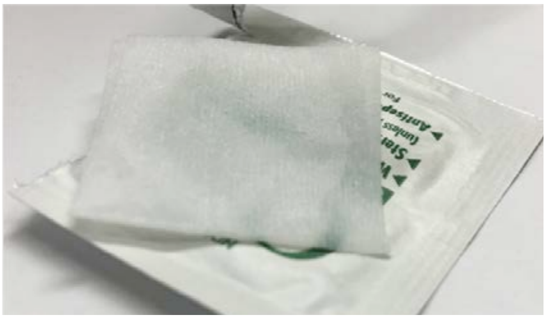

Step 4

Wash your hands and **dry** them with a clean towel.

- Make sure the water is warm, not hot.

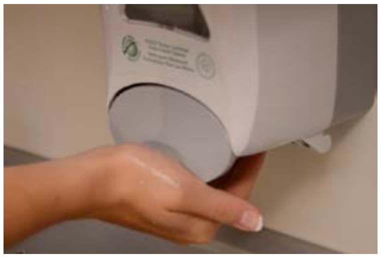

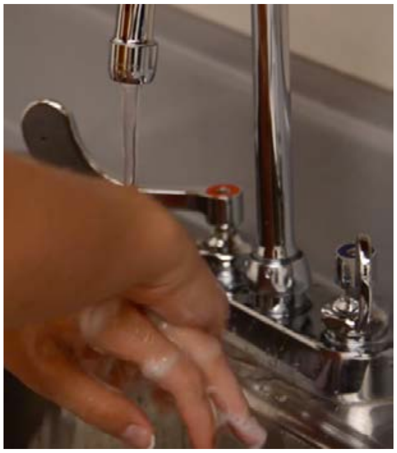

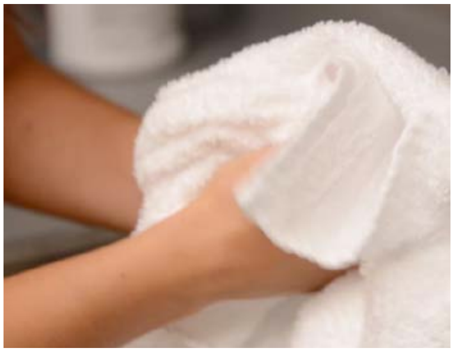

This is very important as it will stimulate blood flow to the hands!

B. PREPARATION

Step 5

Choose a finger to poke on your non-dominant hand.

- If you are left-handed, choose your right hand.
- If you are right-handed, choose your left hand.

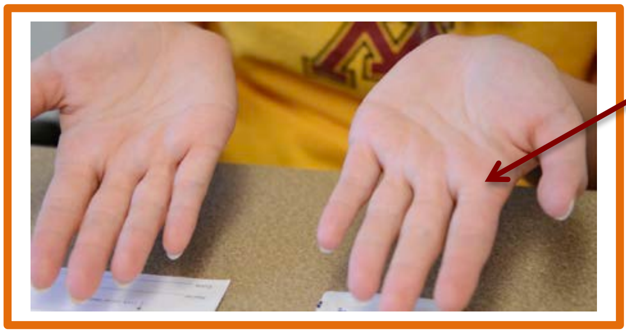

Step 6

Chose an area on the side of your finger to poke.

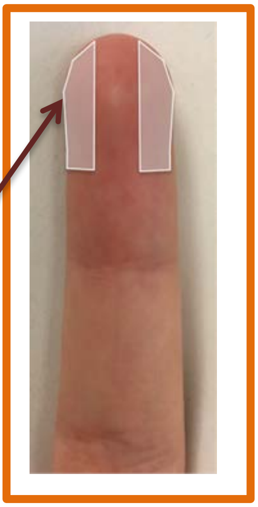

Step 7

Pick up your lancet and twist the cap off.

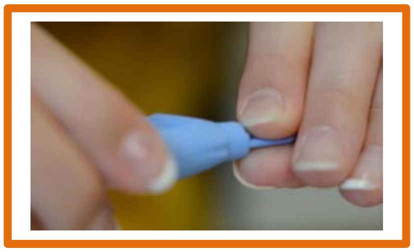

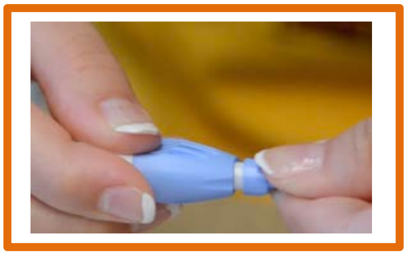

Step 8

Put the lancet down and **wipe the tip** of your finger with the alcohol wipe.

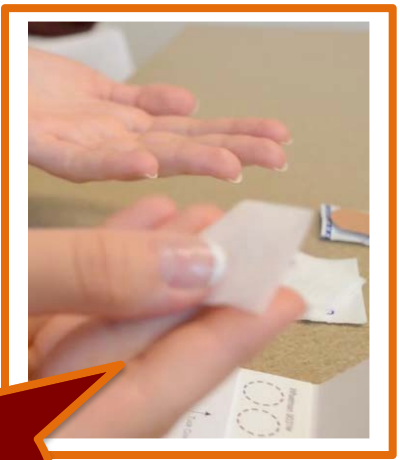

Make sure to only wipe the finger in one single swoop!

C. POKE YOUR FINGER

Step 9

Rub your finger from **base to tip,** and then with your **palm facing up**, press the lancet to your finger until you **hear a click** and feel a slight pinch.

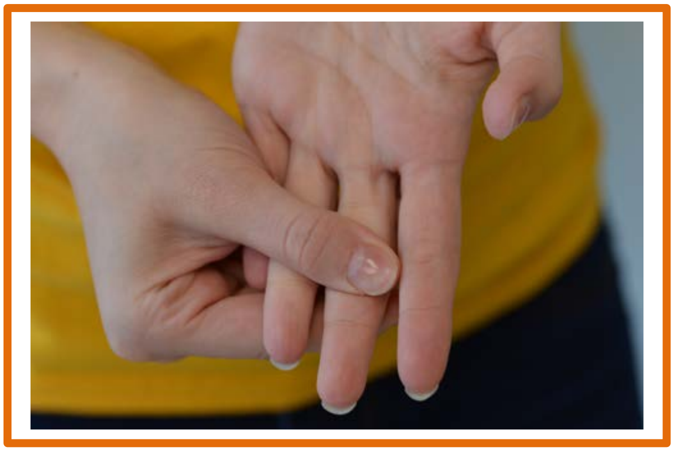
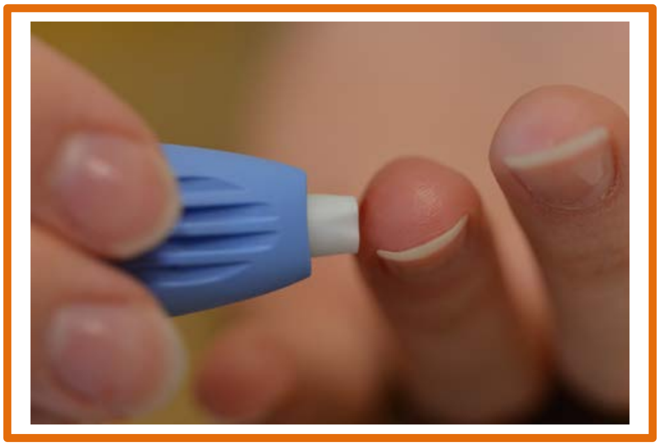
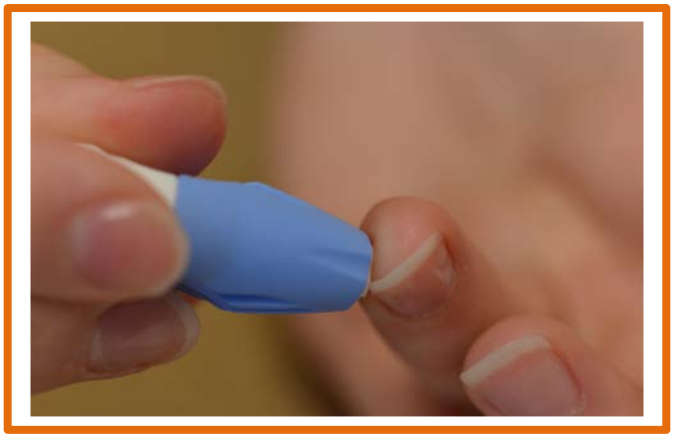
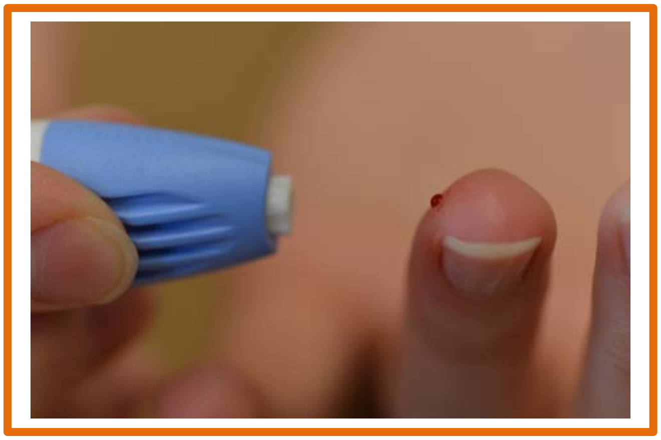

Step 10

Wipe the first drop of blood away with a gauze pad.

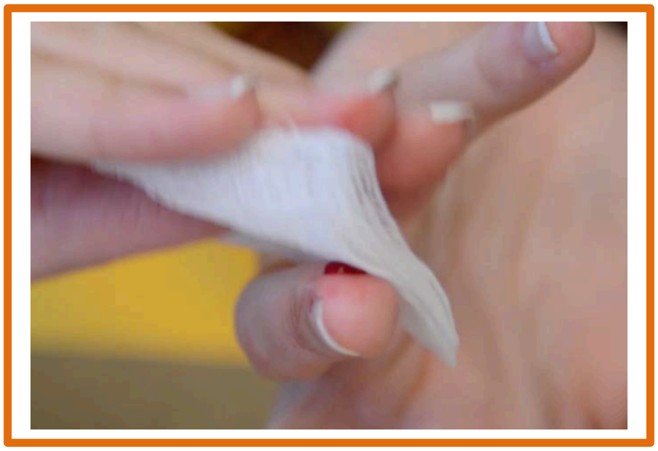

D. COLLECT YOUR SPECIMEN

Step 11

Milk your finger from base to tip to produce more blood.

- **Do not squeeze** your fingertip as this will stop the blood flow.

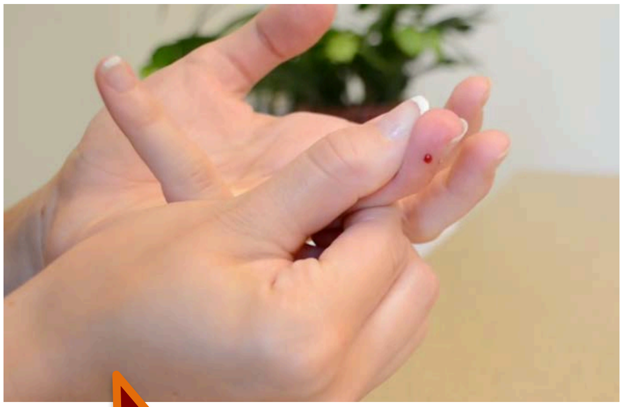

If you're having a difficult time getting blood to flow, <u>try standing up</u> with your hand below your waist.

Step 12

Allow a blood drop to form.

Step 13

Let the blood drop fall from your fingertip into the collection circle.

<u>**DO NOT touch** your finger to the card.</u>

Fill the circle as full as possible, <u>but do not add a second drop of blood to a circle</u>.

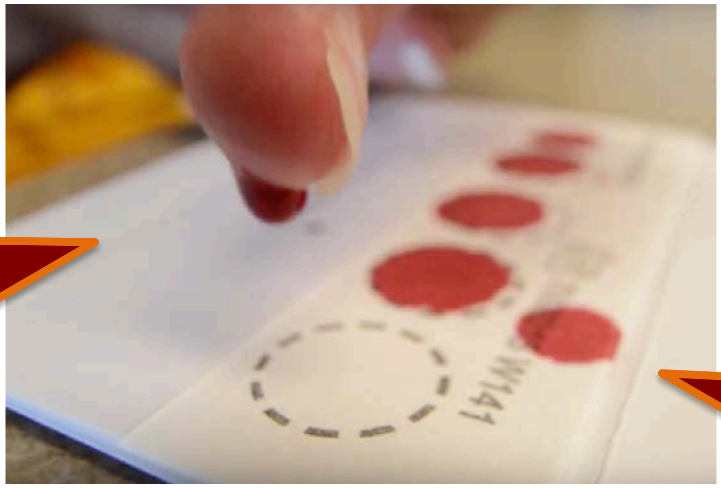

If necessary, use a second lancet!

Examples of <u>good</u> DBS samples

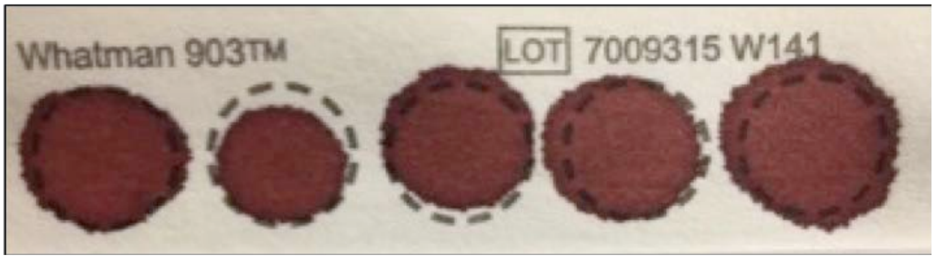

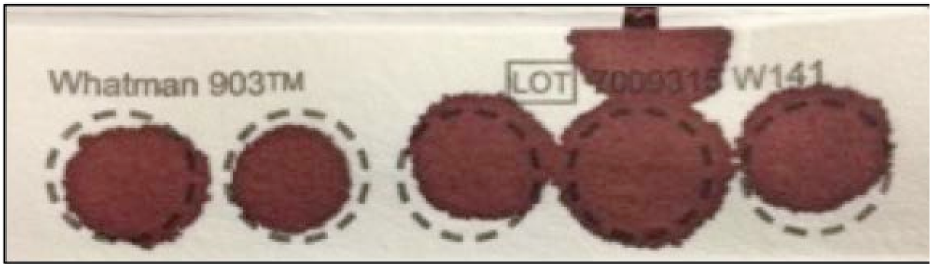

Examples of <u>bad</u> DBS samples

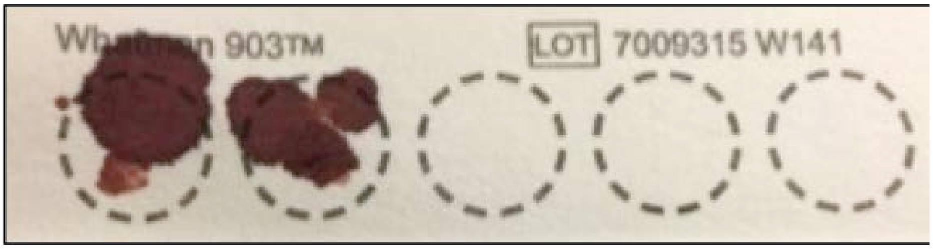

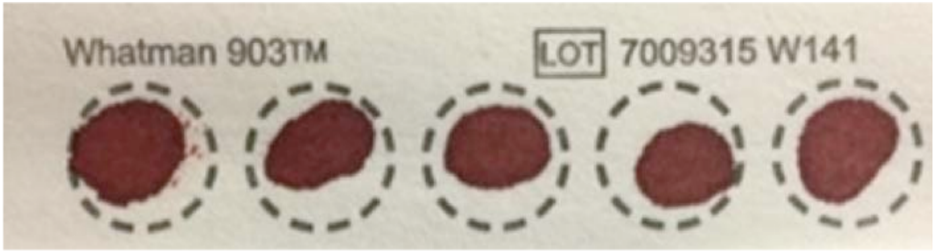

Step 14

After filling in all of the collection card circles, use your **second gauze pad** to wipe the blood**, apply pressure** and **apply a bandage**.

Step 15

Dispose of your alcohol wipe, gauze pad and safety lancet in the trash.

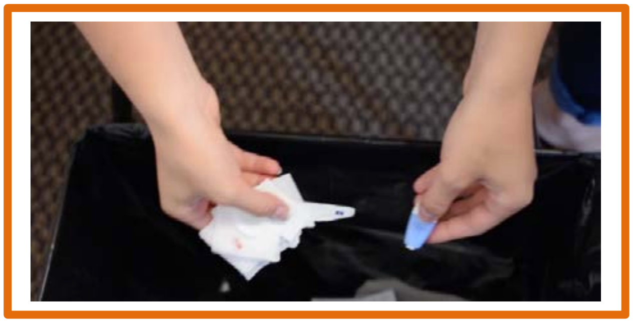

Step 16

Let your collection card **sit for at least 4 hours** to dry before sending it back to us.

- Keep the collection card in a cool, dry place away from sunlight.
- Do not add additional blood drops after blood spots have dried.
- Blood spots will darken as they dry.

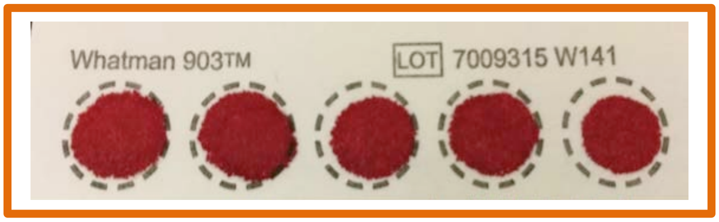

Collection recently completed

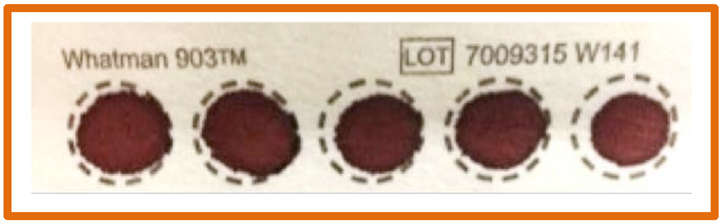

Collection card after drying 4 hours

E. MAIL YOUR SPECIMEN

Step 17

After the blood spots have dried for at least 4 hours, fold the collection card, place it in the **biohazard bag**, and seal it tight**.**

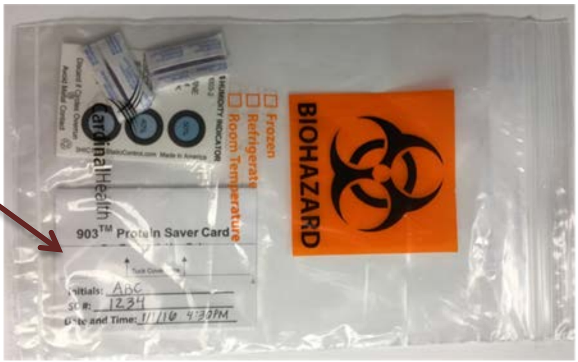

Step 18

Place the plastic bag in the return envelope and seal the envelope.

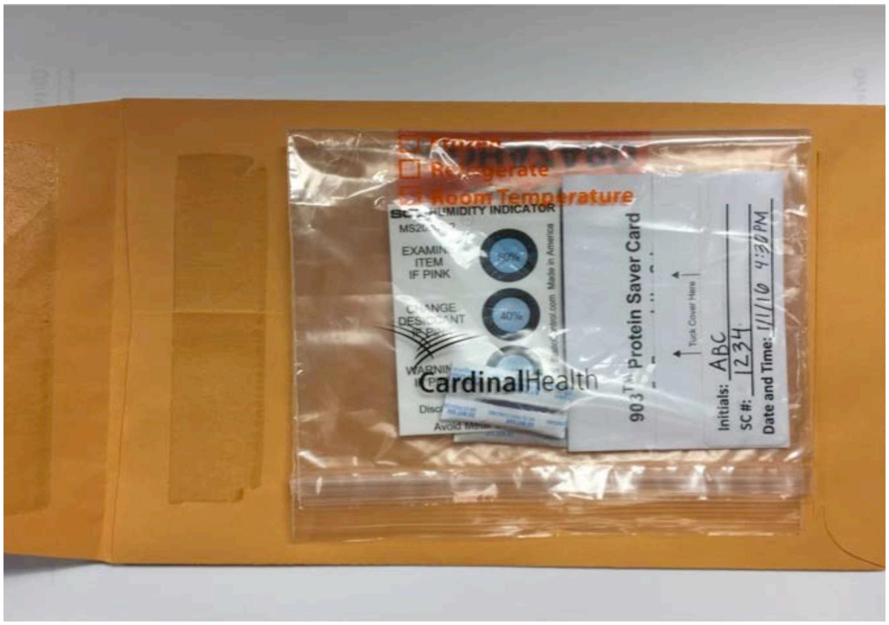

Step 19

Place the return envelope in the mailbox within 24 hours of collection.

F. FREQUENTLY ASKED QUESTIONS

1. Help! How do I know if my blood spots are big enough?

You'll know if you have enough blood if the blood spot fills most of the collection circle. Try your best to fill all five circles.

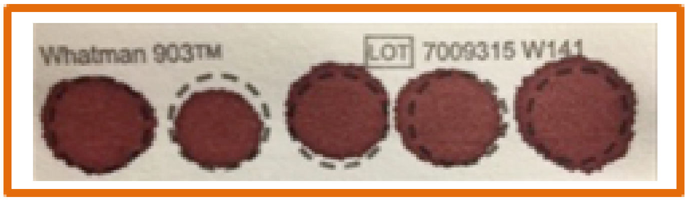

Example of a good collection

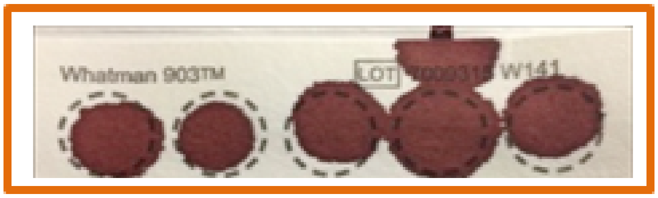

Example of a good collection

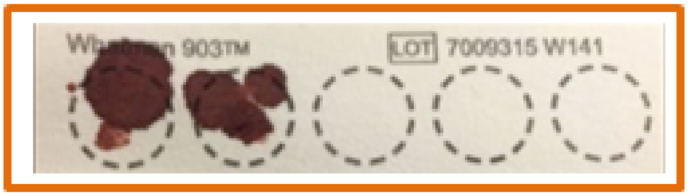

Example of a bad collection
(Multiple drops in one circle and not all circles are filled)

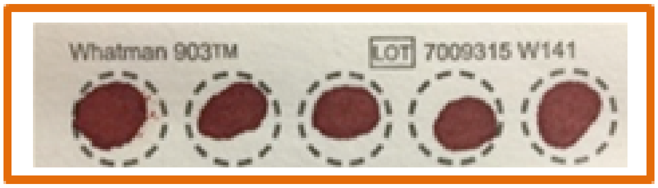

Example of a bad collection
(Spots are too small)

2. My blood drops fell outside of the collection circles. Is this okay?

Yes, it is fine if the blood drops don't fit neatly into the circles, but try to get them as close as possible.

3. I am nervous to poke my finger. What should I do?

Most people have said that the anticipation is worse than the poke itself. It should only feel like a slight "pinch."

4. I poked my finger but am not getting enough blood out. What should I do?

First, try standing up and milking your finger below your waist. You can also try shaking your hand below your waist.

If that doesn't help, you can run your fingers under warm water while milking them to stimulate blood flow.

If blood still isn't flowing, it has probably clotted. Wipe your finger with the alcohol wipe and apply a bandage. Try again later.

It may help to take a break and drink a glass of water and move around. This will help stimulate blood flow to your fingers. Then, try again. This time poke another finger or a different spot on the same finger, and then try the process again.

If you have any questions, please call (612) 624-5081, email dbs@umn.edu or text us at (612) 213-5864.

Thank you for participating in this study!

Dried Blood Spot Collection Instruction Placemat

A. Set-up

Follow steps 1-4 in your instruction booklet. Check the contents of your kit. It should contain these items:

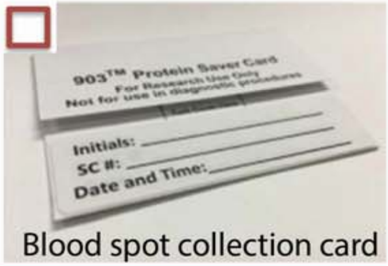

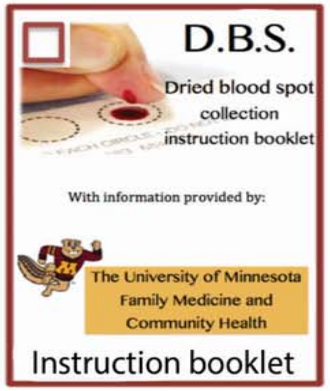

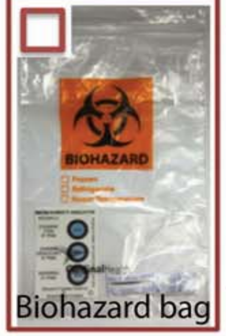

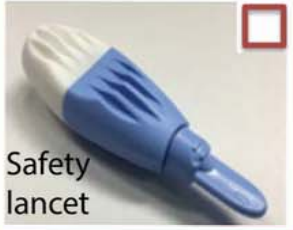

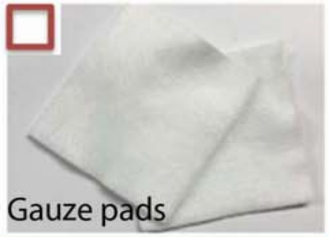

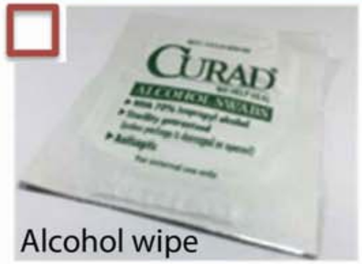

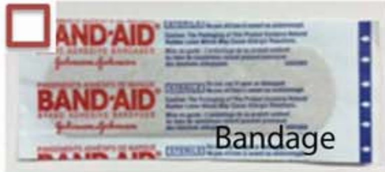

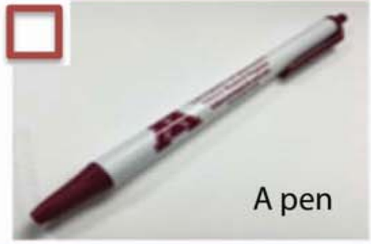

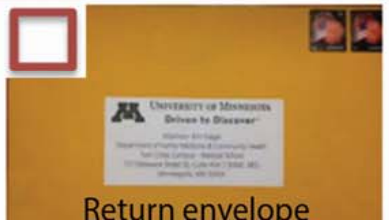

B. Preparation

Follow steps 5-8 in your instruction booklet to prepare your collection.

Place the matching items on their pictures below:

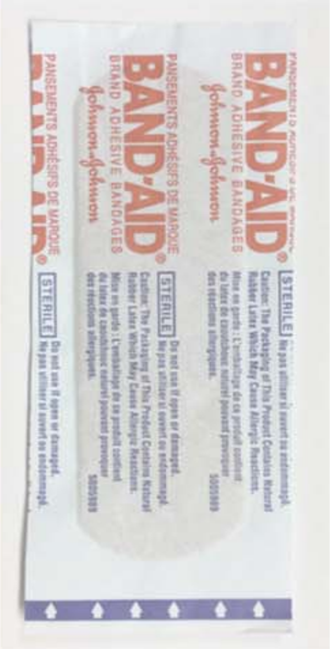

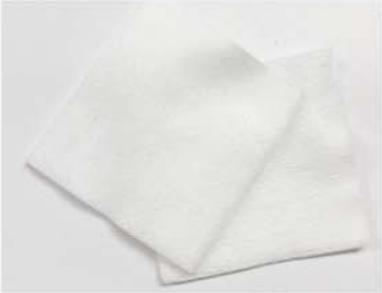

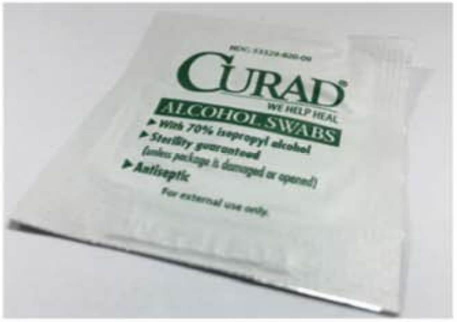

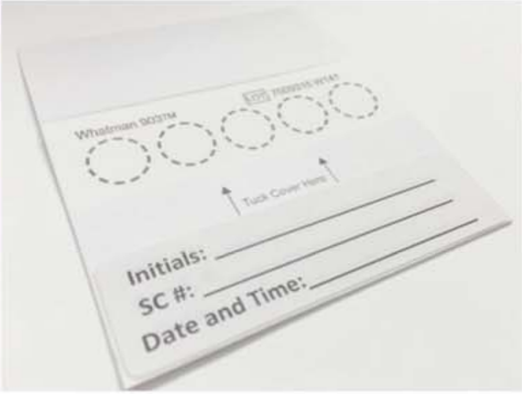

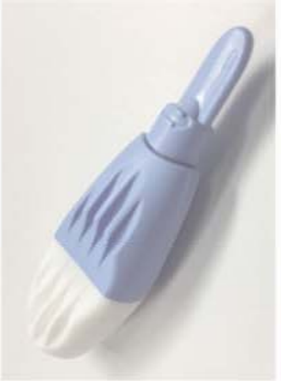

C. Poke your finger

Follow steps 9-10 in the instruction booklet to poke your finger.

D. Collect your specimen

Follow steps 11-16 in the instruction booklet to collect your blood specimen.

Allow your blood spots to dry for at least 4 hours. Do not close the flap on the card while drying.

E. Mail your specimen

Follow steps 17 -19 in the instruction booklet to package and mail your specimen back to us for lab testing. Our address is printed on the return envelope and no additional postage is required for mailing.

If you have any questions, please call (612) 624-5081, email dbs@umn.edu or text us at (612) 213-5864.
